# A Recombinase Polymerase Amplification-Coupled Cas12a Mutant-Based Module for Efficient Detection of Streptomycin-Resistant Mutations in *Mycobacterium tuberculosis*

**DOI:** 10.3389/fmicb.2021.796916

**Published:** 2022-01-06

**Authors:** Peng Liu, Xinjie Wang, Juan Liang, Qian Dong, Jinping Zhang, Dongxin Liu, Shuai Wang, Jing Bi, Wenqi Liu, Zhaoqin Wang, Liang Chen, Lei Liu, Xingxu Huang, Guoliang Zhang

**Affiliations:** ^1^National Clinical Research Center for Infectious Diseases, Guangdong Provincial Clinical Research Center for Tuberculosis, Shenzhen Third People’s Hospital, Southern University of Science and Technology, Shenzhen, China; ^2^Shenzhen Branch, Guangdong Laboratory of Lingnan Modern Agriculture, Genome Analysis Laboratory of the Ministry of Agriculture and Rural Affairs, Agricultural Genomics Institute at Shenzhen, Chinese Academy of Agricultural Sciences, Shenzhen, China; ^3^The Biomedical Translational Research Institute, Faculty of Medical Science, Jinan University, Guangzhou, China; ^4^Department of Laboratory Medicine, Zhongshan Hospital of Sun Yat-sen University, Zhongshan, China; ^5^Intensive Care Unit, Liyuan Hospital, Tongji Medical College, Huazhong University of Science and Technology, Wuhan, China; ^6^Guangdong Center for Tuberculosis Control, Guangzhou, China; ^7^School of Life Science and Technology, ShanghaiTech University, Shanghai, China

**Keywords:** streptomycin, drug resistance, tuberculosis, CRiSPR/Cas, recombinase polymerase amplification

## Abstract

Drug-resistant tuberculosis (TB) is a serious public health problem and threat to global TB prevention and control. Streptomycin (STR) is the earliest and classical anti-TB drug, and it is the earliest drug that generated resistance to anti-TB treatment, which limits its use in treating TB and impedes TB control efforts. The rapid, economical, and highly sensitive detection of STR-resistant TB may help reduce disease transmission and morbimortality. CRISPR/CRISPR-associated protein (Cas) is a new-generation pathogen detection method that can detect single-nucleotide polymorphisms with high sensitivity and good specificity. In this study, a Cas12a RR detection system that can recognize more non-traditional protospacer-adjacent motif-targeting sequences was developed based on Cas12a combined with recombinase polymerase amplification technology. This system detects 0.1% of the target substance, and the entire detection process can be completed within 60 min. Its sensitivity and specificity for detecting clinical STR-resistant *Mycobacterium tuberculosis* were both 100%. Overall, the Cas12 RR detection system provides a novel alternative for the rapid, simple, sensitive, and specific detection of STR-resistant TB, which may contribute to the prompt treatment and prevention of disease transmission in STR-resistant TB.

## Introduction

Tuberculosis (TB), a chronic infectious disease caused by *Mycobacterium tuberculosis* (*M.tb*), is one of the leading causes of death globally (Ranganathan et al., 2018; Gupta-Wright et al., 2019; Liu et al., 2020). According to the World Health Organization (WHO) 2021 Global TB Report, there were approximately 9.9 million new cases and 1.5 million deaths worldwide in 2020. The irrational use of antibiotics causes drug-resistant TB to become more serious (Wang et al., 2017; Tam et al., 2019; World Health Organization (WHO), 2021). In 2020, approximately 157,903 TB patients developed multi-drug resistant TB. Drug-resistant TB is one of the major reasons for high mortality rates and poses an enormous burden on TB prevention and control (World Health Organization (WHO), 2021).

Streptomycin (STR) is the earliest and classical anti-TB drug, but some of its gene locus mutations can lead to drug resistance, which accounted for >60% of all anti-TB drug resistance (Nagai et al., 2013; Smittipat et al., 2016; Sun et al., 2018; Wang et al., 2019; Rocha et al., 2021). Two common gene loci, Lys43Arg, and Lys88Arg in the rpsl gene of STR, have mutation rates as high as 70–90%. Thus, detecting the two site mutations will contribute to diagnosing STR resistance (Wang et al., 2019; Islam et al., 2020).

Currently, the bacteriological examination of drug-resistant TB consists predominantly of *M.tb* drug sensitivity test (DST), GeneXpert MTB/RIF, fluorescent quantitative PCR, DNA sequencing, and whole-genome sequencing (WGS; Hu et al., 2014; Rezaei et al., 2017; Shea et al., 2017; CRyPTIC Consortium and the 100, 000 Genomes Project, Allix-Beguec et al., 2018; Dorman et al., 2018; Sun et al., 2018; Chen et al., 2019; Wang et al., 2019). Among these, DST remains the gold standard for drug-resistant TB in many countries. However, its use is detrimental to the early diagnosis of drug-resistant TB due to its expensive cost, high contamination rate, and time-consuming methodology. Xpert MTB/RIF and fluorescence quantitative PCR require specific instrumentation and high sample quality and do not report specific mutation types. WGS is also relatively expensive, time-consuming, and not universal in many areas. The lack of rapid and sensitive detection technology results in the low positive detection rates of drug-resistant TB. Many drug-resistant TB patients are not being treated promptly, causing disease transmission and deaths. Therefore, the development of a rapid, economical, and highly sensitive detection method is needed to improve the detection rate of drug-resistant TB and help reduce disease transmission and morbimortality (Xu et al., 2020).

CRISPR/Cas is a bacterial adaptive immune system (Tan et al., 2019). Recently, CRISPR/Cas technology has been extensively used because of its speed of detection, simplicity, sensitivity, specificity, and economical benefits (Ai et al., 2019; Kellner et al., 2019; Wang et al., 2020c; Zhang et al., 2021). CRISPR-associated protein (Cas) was used for cleaving foreign nucleic acids through programmable CRISPR-guided RNA (crRNA) due to high nuclease activity (Barrangou et al., 2007; Marraffini and Sontheimer, 2008). Several common subtypes of Cas are Cas9, Cas12, Cas13, and Cas14 (Makarova et al., 2015; Wang et al., 2020b). However, Cas13 protein does not require specific protospacer-adjacent motifs (PAMs) to recognize the target single-stranded RNA but requires *in vitro* transcription to generate the target single-stranded RNA, which is time-consuming and costly. Similarly, the Cas14 protein does not need specific PAMs when it recognizes the target single-stranded DNA, but it needs asymmetric PCR to amplify the target single-stranded DNA, which requires a specific PCR instrument and is time-consuming. CRISPR/Cas12a (CRISPR/Cpf1) belongs to the second family of Cas enzymes (Moreno-Mateos et al., 2017). Cas12a enzyme recognizes PAMs rich in thymine (T) nucleotides, catalyzes its own guidance of crRNA maturation, specifically recognizes and cleaves complementary paired double-stranded DNA (Chen et al., 2018), induces strong non-specific single-stranded DNA (ssDNA) *trans*-cleavage activity, and generates fluorescent signals (Tian et al., 2021). The target double-stranded DNA can be obtained by isothermal amplification, which can be completed without special equipment, takes a short time, and is low in cost. This makes the Cas12a protein widely used in the detection of viruses and bacteria (Ai et al., 2019; Wang et al., 2020c; de Puig et al., 2021).

The Cas12a protein can only recognize traditional PAMs rich in thymine nucleotides, such as the TTTV sequence (V: A, T, G, C) (Zetsche et al., 2015; Chen et al., 2020), considerably limiting its application. Therefore, in this study, two key amino acids at positions 532 and 595 of the Cas12a protein were mutated to G532R and K595R (Toth et al., 2020); called Cas12a RR, this protein can recognize more non-traditional PAM sequences, such as TTCC-, CTCC-, and TCCC-targeting sequences. The Cas12a RR system was applied to detect Lys43Arg (K43R) and Lys88Arg (K88R) nucleic acid sites in *M.tb* rpsl STR-resistant genes.

## Materials and Methods

### Clinical Samples and Ethics Statement

All clinical samples were collected and treated in strict accordance with the WHO-recommended procedure of the Third People’s Hospital of Shenzhen and Center for Tuberculosis Control of Guangdong Province (Guangzhou). All clinical samples were inoculated in 7H10 medium and cultured for 2 weeks. DNA was then extracted from all clinical samples using Bacterial DNA Extraction Kit (Gene Optimal, Shanghai, China) in a BSL-2 laboratory.

### Protein Expression and Purification

The Cas12a RR protein used in the *M.tb* STR-resistant detection system was expressed and purified as previously described (Wang et al., 2020a). The gene encoding Cas12a was codon-optimized, and its 532 and 595 amino acid positions were mutated to G532R and K595R and cloned into the expression vector pET-28a.

### rpsl Gene Primer Designing and Screening

Streptomycin-resistant mutant genes, with reference to the NCBI H37Rv standard strain sequence, were synthesized by Nanjing GenScript, and the names and sequences of the different primers are listed in Supplementary Table 1. A total of 16 pairs of *M.tb* rpsl primers underwent isothermal amplification (recombinase polymerase amplification, RPA) for the *M.tb* rpsl gene, which was used to screen primers with a better amplification effect. The amplified *M.tb* rpsl genes were then added to the Cas12a reaction system. After incubation at 37°C for 15 min, fluorescence results were observed using a microplate reader and *via* naked eye detection under fluorescent light. The primer corresponding to the strongest fluorescent signal was selected for subsequent experiments.

### crRNA Designing and Optimization

According to the *M.tb* rpsl wild-type gene and rpsl K43R and rpsl K88R STR-resistant mutant genes, we designed a crRNA, with a length of 23 bp, that covered the mutation site, and the specific crRNA was located behind the PAM (Chen et al., 2018, 2020; Kellner et al., 2019). Meanwhile, a mismatched base at the non-mutation site of crRNA was designed to reduce the interference signal caused by the *M.tb* rpsl wild-type gene. After the design was completed, crRNA was synthesized by GenScript, and the names and core sequences of different crRNAs are listed in Supplementary Table 2.

### Isothermal Amplification

The RPA of the rpsl gene was performed with a commercial ERA kit (Suzhou GenDx Biotech Co., Ltd., Jiangsu, China), following the instructions from the manufacturer. Briefly, a 50-μl reaction containing 2 μl DNA sample, 2 μl forward primer (10 μM), 2 μl reverse primer (10 μM), and 2.5 μl magnesium acetate (280 mM) was incubated at 37°C for 30 min. The RPA reaction was then transferred to the Cas12a cleavage assay.

### CRISPR/Cas12a Detection Reaction

Detection assays were performed with 200 ng purified Cas12a RR protein, 25 pM ssDNA FQ probe sensor, 1 μM crRNA, and 2 μl sample DNA in a reaction buffer (100 mM NaCl, 50 mM Tris–HCl, 10 mM MgCl_2_, and 100 μg/ml BSA, pH 7.5) in a 20-μl reaction volume at 37°C. A microplate reader (Thermo Fisher Scientific) was used for fluorescence intensity detection. Fluorescence kinetics was monitored using a monochromator, with excitation at 485 nm and emission at 520 nm. At the same time, green fluorescence was observed by the naked eye under 485-nm light.

### Statistical Analysis

All experimental results are shown as mean ± SEM unless stated otherwise. When only two groups were compared, statistical significance was assessed using an unpaired Student’s *t*-test. Significance was indicated as ^∗^*p* < 0.05, ^∗∗^*p* < 0.01, ^∗∗∗^*p* < 0.001, and ^∗∗∗∗^*p* < 0.0001. Statistical analyses were conducted using GraphPad Prism 7.0.

## Results

### Cas12a RR Mutant Protein Can Recognize Non-traditional Targeting Sequences

The Cas12a protein can only recognize the traditional TTTV target sequence. Therefore, the prokaryotic codon for the Cas12a protein–nucleic acid sequence was optimized; the 532 and 595 amino acids sites were mutated to G532R and K595R and inserted into the pET28a expression vector, which induced soluble protein expression at a low temperature. The target protein, Cas12a RR, was obtained *via* affinity purification and molecular sieve purification (Supplementary Figure 1).

**FIGURE 1:**
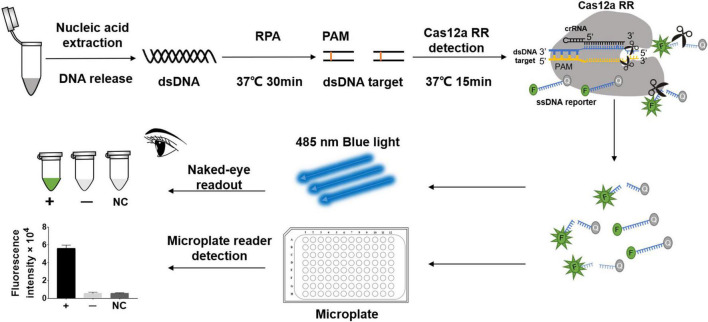
Schematic diagram of CRISPR/Cas12a RR in detecting streptomycin (STR)-resistant *Mycobacterium tuberculosis*. Schematic diagram of Cas12a RR-mediated STR-resistant *M.tb* genome detection. Specific CRISPR-guided RNAs (crRNAs) targeting the *M.tb* rpsl gene were designed for STR-resistant *M.tb* detection. When CRISPR/Cas12a RR proteins cleave double-stranded DNA with the specific crRNA guide, they induce robust and non-specific single-stranded DNA (ssDNA) *trans*-cleavage. The ssDNA (sequence TTATT) reporter fluoresces when the quenched fluorescent ssDNA is cleaved (Chen et al., 2018; Xiao et al., 2020). F, fluorophore; Q, quencher.

**FIGURE 2:**
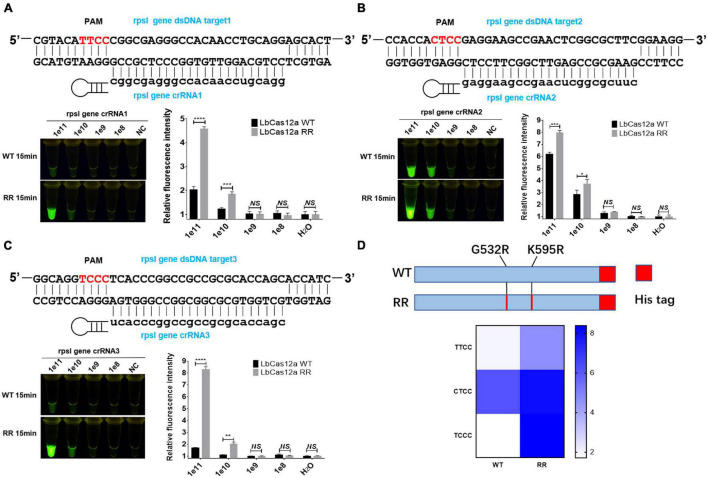
Functional verification of the Cas12a RR protein. The Cas12a RR protein recognizes the TTCC **(A)**, CTCC **(B)**, and TCCC **(C)** targeting sequences. The fluorescent images and fluorescence intensity **(A–C)** at 15 min of the reaction are shown. **(D)** Cas12a RR protein recognizes TTCC, CTCC, and TCCC target sequence heat map. Data are presented as the mean ± SEM from at least three independent experiments. *p < 0.05, **p < 0.01, ***p < 0.001, and ****p < 0.0001.

The rpsl gene fragment of *M.tb* contains TTCC, CTCC, and TCCC non-traditional targeting sequences that could be recognized by programmable crRNA. Therefore, the rpsl gene and crRNA were added to the Cas12a wild-type and Cas12a RR systems, respectively, after having been allowed to react for 15 min at 37°C. Conversely, a full-wavelength microplate reader was used to detect fluorescence values with 485-nm excitation wavelength and 520-nm emission wavelength. Two systems were placed under 485-nm laser light, and the results were assessed based on fluorescence intensity determined by the naked eye (Figure 1). When crRNA specifically recognized the target nucleic acid fragment, the product solution caused a color change from colorless to fluorescent green. In contrast, if there was no target gene that matched crRNA, then the reaction product solution remained colorless. The results showed that the detection effect of the Cas12a RR system is significantly higher than that of the Cas12a wild type (Figures 2A–D; ^∗∗∗∗^*p* < 0.0001, Figure 2A; ^∗∗∗^*p* < 0.001, Figure 2B; ^∗∗∗∗^*p* < 0.0001, Figure 2C), regardless of whether the result was from a microplate reader or naked eye. The Cas12a RR protein had a good detection effect for non-traditional targeting sequences, such as TTCC (Figure 2A, ^∗∗∗∗^*p* < 0.0001), CTCC (Figure 2B, ^∗∗∗^*p* < 0.001), and TCCC (Figure 2C, ^∗∗∗∗^*p* < 0.0001), other than TTTV; therefore, it was used for subsequent detection.

### Screening and Optimization of the crRNA at rpsl K43R and rpsl K88R Streptomycin-Resistant Mutation Sites

**FIGURE 3:**
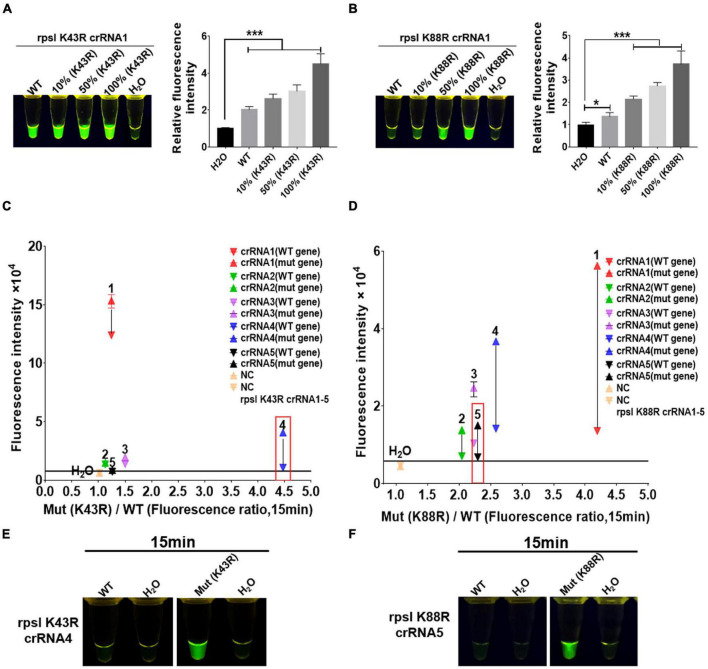
Screening and optimization of the crRNA at rpsl K43R and rpsl K88R streptomycin (STR)-resistant mutation sites. The Cas12a RR system detects the efficiency of the rpsl gene-specific crRNA of STR-resistant *M.tb*. The fluorescent images and intensities **(A,B)** at 15 min of the reaction are shown. The rpsl gene fragments of wild-type *M. tuberculosis* and streptomycin-resistant *M. tuberculosis* (K43R or K88R) were mixed in different proportions to obtain the detection samples with mutation rates of 100, 50, and 10%, respectively. **(C,D)** Specificity was determined as the ratio of the on-target DNA-induced fluorescence intensity over the off-target DNA-induced fluorescence intensity. The fluorescence intensity was obtained from crRNA–Cas12a RR reaction for 15 min and measured using a microplate reader. The core crRNA sequences are listed in Supplementary Table 2. **(E,F)** The Cas12a RR system verifies the specific crRNA of the STR-resistant *M.tb* rpsl gene fragment and shows the fluorescence image at 15 min of reaction. The concentration of the target gene used in the above-mentioned detection reaction is 1 × 10^11^ copies. Data are presented as the mean ± SEM from at least three independent experiments. *p < 0.05, **p < 0.01, ***p < 0.001, and ****p < 0.0001.

crRNAs, 23 bp in length, were designed according to the non-traditional targeting sequence of the *M.tb* rpsl wild-type gene and rpsl K43R and rpsl K88R STR-resistant mutant genes. The Cas12a RR detection system was used to detect the efficiency of crRNA. The results showed interference signals when rpsl K43R- and rpsl K88R-specific crRNA recognized *M.tb* wild-type genes (Figures 3A,B). To further reduce the interference signal of *M.tb* wild type genes, one mismatch base was designed at the non-mutation site on the specific crRNA. The results were assessed according to the intensity of fluorescence by two methods: microplate reader and naked eye. The results showed that K43R crRNA4, K88R crRNA5 has better specificity, and it was selected for subsequent experiments (Figures 3C–F and Supplementary Figures 2, 3).

### Primers of *Mycobacterium tuberculosis* rpsl Gene Fragment Screened *via* Recombinase Polymerase Amplification

**FIGURE 4:**
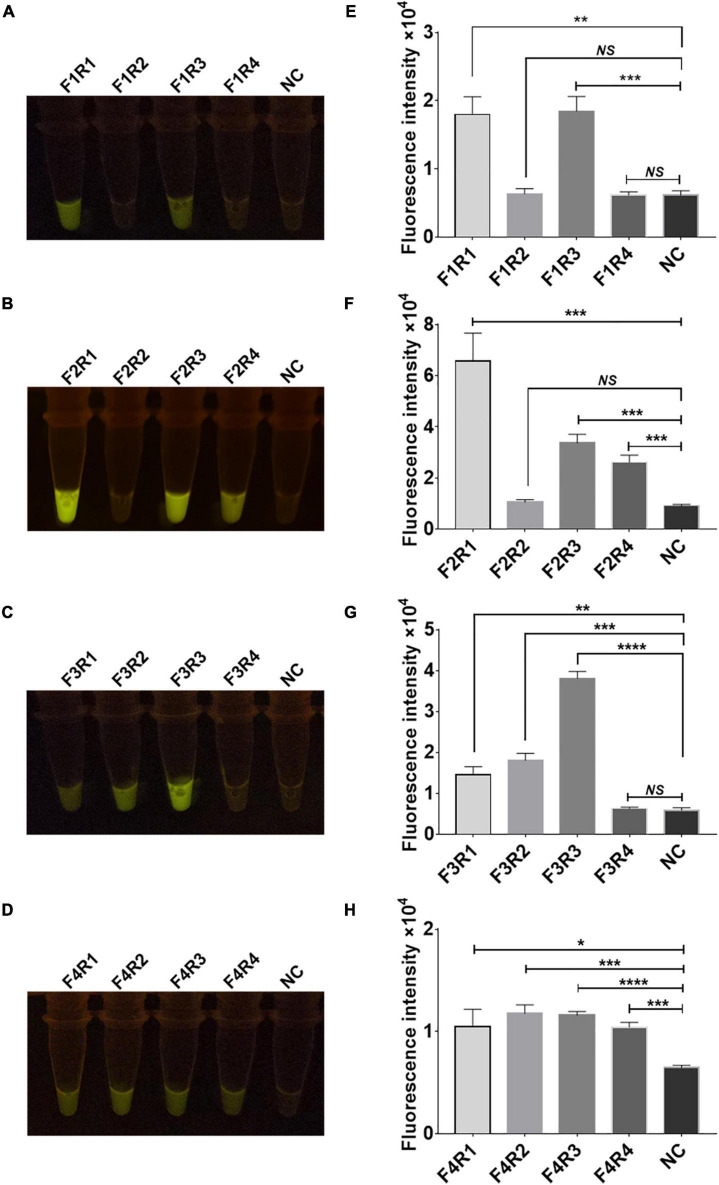
Screening of recombinase polymerase amplification (RPA) primers for the rpsl gene of streptomycin (STR)-resistant *M. tuberculosis*. Optimization of high-efficiency RPA primers used to amplify the rpsl-related gene fragments of STR-resistant *M.tb*. The amplification efficiency of 16 pairs of RPA primers was evaluated; the fluorescent images **(A–D)** and fluorescence intensity **(E–H)** at 15 min of reaction are shown. The content of the STR-resistant *M.tb* whole-genome template used in the above-mentioned detection reaction is 100 ng. Data are presented as the mean ± SEM from at least three independent experiments. *p < 0.05, **p < 0.01, ***p < 0.001, and ****p < 0.0001.

Sixteen pairs of *M.tb* rpsl primers underwent RPA for the *M.tb* rpsl gene. The RPA products were added to the Cas12a RR detection system and incubated for 15 min at 37°C. The results were assessed according to the intensity of fluorescence by the two methods: naked eye (Figures 4A–D) and microplate reader (Figures 4E–H). The results show that the F2R1 primers have a good amplification effect (Figures 4B,F). This pair of primers was used in subsequent detection experiments.

### High Sensitivity and Specificity Detection of rpsl K43R and rpsl K88R Streptomycin-Resistant Mutation Sites Based on Cas12a RR Fluorescence Method

**FIGURE 5:**
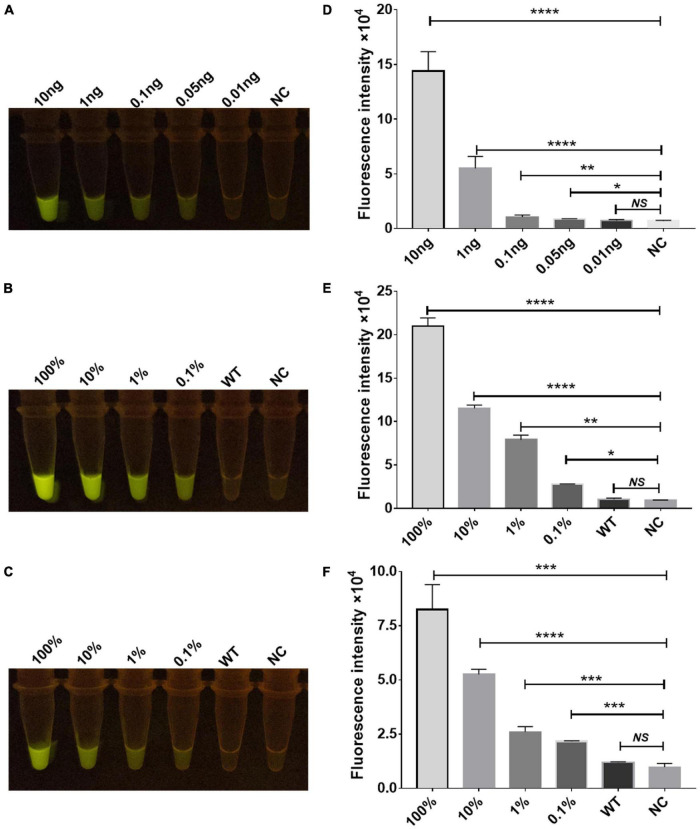
Sensitivity and specificity verification of the Cas12a RR detection system. Sensitivity assay using rpsl-crRNAmix to detect gradient-diluted rpsl gene RNA from 10 to 0.01 ng **(A,D)**. Specificity **(B,C,E,F)** of the Cas12a RR fluorescence method for detecting the rpsl gene of streptomycin-resistant *M.tb.* The wild-type *M. tuberculosis* and streptomycin-resistant *M. tuberculosis* genome nucleic acids were mixed in different proportions to obtain test samples with mutation rates of 100, 10, 1, 0.1, and 0%. The fluorescent images **(A–C)** and fluorescence intensity **(D–F)** at 15 min of reaction are shown. Data are presented as the mean ± SEM from at least three independent experiments. *p < 0.05, **p < 0.01, ***p < 0.001, and ****p < 0.0001.

To determine the sensitivity of the Cas12a RR fluorescence method for the detection of STR-resistant *M.tb*, RPA was performed to amplify the rpsl gene fragment of STR-resistant *M.tb*. Two microliters of RPA product was added to the 20-μl Cas12a RR system and mixed well. After incubation for 15 min at 37°C, fluorescence intensity was determined by the naked eye (Figures 5A–C) and the microplate reader (Figures 5D–F). The results showed that RPA can amplify an STR-resistant fragment from 0.05 ng *M.tb* whole genome with high sensitivity (Figures 5A,D). Meanwhile, to detect whether the Cas12a RR fluorescence method has a high specificity to effectively distinguish the rpsl wild-type gene and rpsl K43R and rpsl K88R STR-resistant mutant genes of *M.tb*, the whole genome of *M.tb* wild type was mixed with STR-resistant mutant genes in different proportions to obtain test samples with mutation rates of 100, 10, 1, 0.1, and 0%. A 1-μl sample from each group was used as a template. Water served as a negative control and was added to the 50-μl RPA system; the mixture was incubated at 37°C for 30 min. Subsequently, 2 μl of RPA products was added to the 20-μl Cas12a RR detection system. After reaction at 37°C for 15 min, fluorescence intensity was assessed using the microplate reader and the naked eye. The results showed that the Cas12a RR fluorescence method can detect 0.1% rpsl K43R (Figures 5B,E) and rpsl K88R (Figures 5C,F) STR-resistant mutant genes. The above-mentioned results suggested that the Cas12a RR fluorescence method has high sensitivity and excellent specificity for the detection of *M.tb* rpsl K43R and rpsl K88R STR-resistant mutation sites.

### Cas12a RR System Rapid Detection of Clinical Streptomycin-Resistant Strains

**FIGURE 6:**
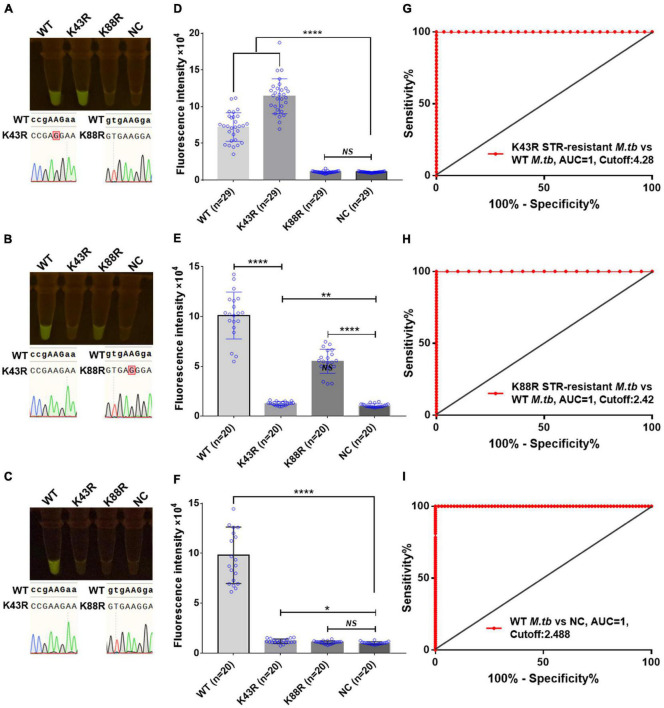
Cas12a RR detects the clinical culture strains of streptomycin (STR)-resistant *M. tuberculosis*. The fluorescent images **(A–C)** and fluorescence intensity **(D–F)** at 15 min of reaction are shown. Diagnostic performance of Cas12a RR fluorescence system **(G–I)**. **(G)** Receiver operating characteristic (ROC) curve of the fluorescence value of K43R STR-resistant *M.tb vs*. WT *M.tb* control group. **(H)** ROC curve of the fluorescence value of K88R STR-resistant *M.tb vs*. WT *M.tb* control group. **(I)** ROC curve of the fluorescence value of WT *M.tb vs*. NC control group. Cutoff values were determined using Youden’s index, *i*.*e*., the maximum of (sensitivity + specificity – 1). The content of the whole-genome template of the clinical sample used in the above-mentioned detection reaction is 1 ng. Data are presented as the mean ± SEM from at least three independent experiments. *p < 0.05, **p < 0.01, ***p < 0.001, and ****p < 0.0001.

Furthermore, we conducted a verification in clinical STR-resistant *M.tb* using the Cas12a RR system. Forty-nine clinical STR-resistant TB samples were collected and cultured for 2 weeks. DNA was extracted. One nanogram of DNA from the above-mentioned samples was separately added to the 50-μl RPA reaction system, mixed well, and incubated at 37°C for 30 min. Next, 2-μl of reaction products was added to the Cas12a RR detection system to obtain the total volume of 20 μl. After mixing well, the reaction was incubated at 37°C for 15 min. The results were then analyzed *via* the detection of fluorescence intensity by the naked eye (Figures 6A–C) and microplate reader (Figures 6D–F). Both methods can detect and distinguish rpsl K43R (Figures 6A,D,G) and rpsl K88R STR-resistant *M.tb* (Figures 6B,E,H) with consistent results. Compared with DNA sequencing experiments, the sensitivity and specificity of the Cas12a RR system for detecting STR-resistant strains were both 100% (Figures 6G–I and Tables 1, 2), and the two methods have high consistency.

**TABLE 1:** Performance comparison between CRISPR/Cas12a RR and DNA sequencing for streptomycin (STR)-resistant (K43R) detection in clinical samples.

Methods	CRISPR/Cas12a RR			Comparison of two methods		
DNA sequencing	Positive	Negative	Total	Sensitivity (%)	Specificity (%)	Kappa
Positive	29	0	29	100	100	1.00
Negative	0	20	20			
Total	29	20	49			

*K43R, Lys43Arg nucleic acids sites in M.tb rpsl STR-resistant gene.*

**TABLE 2:** Performance comparison between CRISPR/Cas12a RR and DNA sequencing for STR-resistant (K88R) detection in clinical samples.

Methods	CRISPR/Cas12a RR			Comparison of two methods		
DNA sequencing	Positive	Negative	Total	Sensitivity (%)	Specificity (%)	Kappa
Positive	20	0	20	100	100	1.00
Negative	0	20	20			
Total	20	20	40			

*K88R, Lys88Arg nucleic acids sites in M.tb rpsl STR-resistant gene.*

## Discussion

At present, DST of *M.tb* is still the gold standard for the diagnosis of drug-resistant pulmonary TB in many countries (Rezaei et al., 2017; Shea et al., 2017; Wang et al., 2019). However, due to the expensive equipment, high contamination rates, and time-consuming methodology, it is not conducive for the early diagnosis of diseases. The low positive detection rate of drug-resistant TB and lack of rapid and sensitive detection methods have prevented the majority of drug-resistant TB patients from receiving prompt treatment. This has resulted in an increase in the number of deaths through an increase in disease transmission, which cause many difficulties for the prevention and control of TB. Therefore, a simple, rapid, and high-sensitivity method for detecting drug-resistant TB is critically needed.

In this study, we developed a rapid fluorescence detection method based on the Cas12a RR system. Using this system, we selected the rpsL gene to identify whether the samples contained STR-resistant *M.tb*. At the same time, we selected Lys43Arg and Lys88Arg of the rpsL gene to determine whether the samples contained *M.tb* STR-resistant DNA. The whole detection process was completed in 60 min without complicated instruments. More importantly, the test results were visible to the naked eye, which will help to reduce the cost of professional training for the operators.

The Cas12 enzyme used in SHERLOCK requires a PAM sequence for cleavage at the target site (Kellner et al., 2019; Chen et al., 2020). As there is no traditional TTTV-targeting sequence recognized by the Cas12a protein near the mutation site of the *M.tb* STR-resistant gene, we constructed the Cas12a RR mutant protein to recognize at least TTCC, CTCC, and TCCC sequences other than TTTV (Toth et al., 2020). This allows the Cas12a RR detection system to detect the *M.tb* STR-resistant gene because rpsl K43R- and rpsl K88R-specific crRNAs have interference signals in recognizing wild-type genes. Therefore, a mismatch base was designed at the non-target site on the crRNA to design a crRNA with single-base-pair specificity. When the target base pair does not exist, this synthetic mismatch will produce a base pair bubble, resulting in more stringent recognition at the target location (Kellner et al., 2019). Finally, we successfully identified the specific crRNA that can recognize the wild-type gene without an interference signal within 15 min. Single-base-pair differences in target sequences can be distinguished by the specific crRNA. However, if the target nucleic acid content in the detection system is too high or the reaction time is too long, it will cause the Cas12a RR enzyme to generate interference signals when detecting wild-type genes. Therefore, we reduced the target nucleic acid content in the detection system and maintained the detection time within 15 min; we even completed the detection in a shorter time. In this way, the generation of interference signals can be avoided.

One of the attractive features of Sherlock is the rapidity of the experiments (Kellner et al., 2019). Compared with traditional PCR, RPA can control the nucleic acid amplification time within 30 min, which simplifies the nucleic acid amplification process and shortens the amplification time (Chen et al., 2018; Gootenberg et al., 2018; Ai et al., 2019; Kellner et al., 2019). In this study, the target gene was amplified from the whole 0.05 ng *M.tb* genome *via* RPA and was recognized by Cas12a RR. At the same time, the single-nucleotide specificity of Sherlock has also been applied to mutations associated with *M.tb* STR resistance with high sensitivity and specificity, and even if the target gene content is 0.1% of the total sample volume, the gene can be recognized by Cas12a RR (Kellner et al., 2019).

After establishing the Cas12a RR detection method in *M.tb* STR resistance, we evaluated its diagnostic value in clinical STR-resistant TB patients. Compared with the existing conventional diagnostic methods, the Cas12a RR method has the advantages of short sample processing time, high detection rate (100%), and acceptable performance in terms of sensitivity (100%) and specificity (100%). This study shows that the Cas12a RR method is highly effective and feasible for the diagnosis of STR-resistant TB, which lays the foundation for the application of the Cas12a RR method in diagnosing STR-resistant TB. However, a major limitation of our study is that we only conducted verification on a small number of clinically cultured strains of STR-resistant *M.tb*. In addition, *M.tb* STR resistance is mainly caused by mutations in the three genes – rpsL, rrs, and gidB (Wang et al., 2019; Islam et al., 2020). Whereas our study only detected the STR-resistant *M.tb* strains caused by rpsL gene mutation, the STR-resistant *M.tb* strains caused by mutations in rrs and gidB have not been tested.

## Conclusion

In summary, this study proposes for the first time a highly sensitive diagnostic method for detecting STR-resistant TB based on the Cas12a system, which was further evaluated in clinical isolates of STR-resistant TB patients. Compared with drug susceptibility experiments and genome sequencing, the Cas12a detection system has higher sensitivity and specificity, and the entire detection process can be completed within 60 min, which is also more advantageous in sample processing (Wang et al., 2020a,c; Meng et al., 2021). This brings hope for the timely diagnosis and treatment of STR-resistant TB patients. Our research shows the excellent diagnostic value of using the Cas12a system in detecting STR-resistant TB. Therefore, it is necessary to use high-throughput screening to test more clinically cultured strains of STR-resistant *M.tb* and sputum specimens from patients with STR-resistant tuberculosis to further verify the results of this study.

## Data Availability Statement

The original contributions presented in the study are included in the article/Supplementary Material, further inquiries can be directed to the corresponding authors.

## Ethics Statement

The studies involving human participants were reviewed and approved by the Shenzhen Third People’s Hospital, Southern University of Science and Technology, Shenzhen, China. The patients/participants provided their written informed consent to participate in this study.

## Author Contributions

PL: conceptualization. PL and JL: data curation and writing – original draft. XW: formal analysis and methodology. XH and GZ: funding acquisition and project administration. XW, DL, SW, JB, WL, LC, XH, and GZ: resources. ZW, LL, XH, and GZ: supervision. JZ: validation. PL and QD: visualization. All authors contributed to the article and approved the submitted version.

## Conflict of Interest

The authors declare that the research was conducted in the absence of any commercial or financial relationships that could be construed as a potential conflict of interest.

## Publisher’s Note

All claims expressed in this article are solely those of the authors and do not necessarily represent those of their affiliated organizations, or those of the publisher, the editors and the reviewers. Any product that may be evaluated in this article, or claim that may be made by its manufacturer, is not guaranteed or endorsed by the publisher.
