# Supplementary material for: A Recombinase Polymerase Amplification-Coupled Cas12a Mutant-Based Module for Efficient Detection of Streptomycin-Resistant Mutations in Mycobacterium tuberculosis
Source: Front Microbiol. 2022 Jan 6;12:796916. doi: 10.3389/fmicb.2021.796916 (PMC8770913; doi:10.3389/fmicb.2021.796916)
Supplement: Supplementary file 1 [file Data_Sheet_1.docx]

**Supplementary Tables**

**Supplementary Table 1. Primers used in this study.**

| Primer name | Primer sequence |
| --- | --- |
| rpsl F | atgccaaccatccagcag |
| rpsl R | tcagcccttctccttcttagc |
| rpsl RPA-F1 | tcgtcgggacaagatcagtaaggtcaagaccgcg |
| rpsl RPA-F2 | tcagtaaggtcaagaccgcggctctgaagggc |
| rpsl RPA-F3 | tctgaagggcagcccgcagcgtcgtggtgtatgc |
| rpsl RPA-F4 | ctctgaagggcagcccgcagcgtcgtggtgtatgcacccg |
| rpsl RPA-R1 | cctgtttgcggttcttgacaccctgcgtatccagcg |
| rpsl RPA-R2 | ttcttgacaccctgcgtatccagcgaaccgcgg |
| rpsl RPA-R3 | tatccagcgaaccgcggatgatcttgtagcgc |
| rpsl RPA-R4 | tccagcgaaccgcggatgatcttgtagcgcacaccaggc |

RPA: recombinase polymerase amplification.

**Supplementary Table 2. Core sequences of synthetic mismatched crRNA.**

| crRNA name | crRNA sequence | |
| --- | --- | --- |
| rpsl WT-crRNA1 | cggcgagggccacaaccUgcagg | |
| rpsl K43R-crRNA1 | UcggagUggUggUgUacacgcgg | |
| rpsl K43R-crRNA2 | UcggacUggUggUgUacacgcgg | |
| rpsl K43R-crRNA3 | UcgcagUggUggUgUacacgcgg | |
| rpsl K43R-crRNA4 | gaggaagccgaacUcggcgcUUc | |
| rpsl K43R-crRNA5 | gagUaagccgaacUcggcgcUUc | |
| rpsl K88R-crRNA1 | Ucacccggccgccgcgcaccagc | |
| rpsl K88R-crRNA2 | | UcacccggccgccgcgUaccagc |
| rpsl K88R-crRNA3 | | UcacccggccgccgcgcUccagc |
| rpsl K88R-crRNA4 | | UcacccggccgccgcgcaUcagc |
| rpsl K88R-crRNA5 | | Ucacccggccgccgcacaccagc |

crRNA: CRISPR guided-RNA.
