# Supplementary material for: A Recombinase Polymerase Amplification-Coupled Cas12a Mutant-Based Module for Efficient Detection of Streptomycin-Resistant Mutations in Mycobacterium tuberculosis
Source: Front Microbiol. 2022 Jan 6;12:796916. doi: 10.3389/fmicb.2021.796916 (PMC8770913; doi:10.3389/fmicb.2021.796916)
Supplement: Supplementary file 2 [file Image_1.pdf]

Supplementary Figures

Supplementary Figure 1

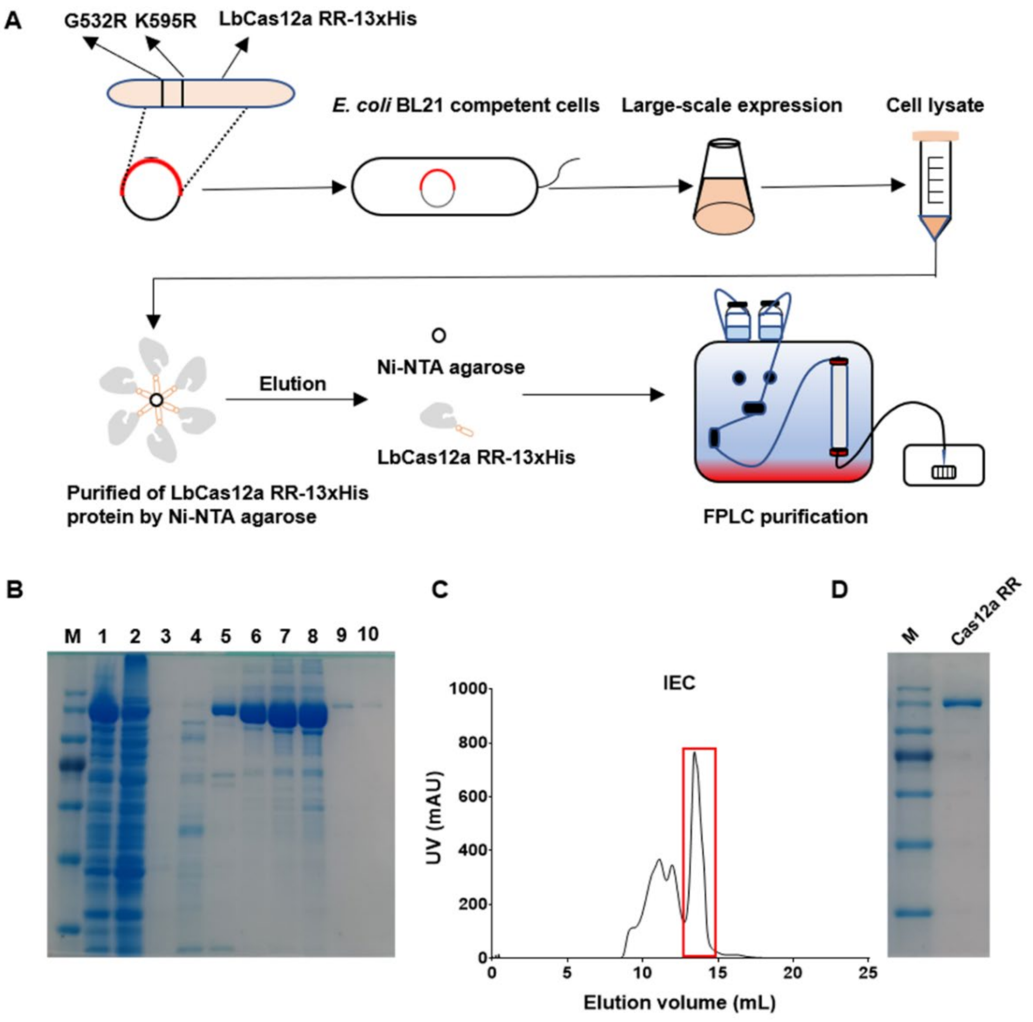

Supplementary Figure 1. Expression and purification of Cas12a RR protein

(A) Cas12a RR protein expression and purification process. First, the Cas12a RR protein expression plasmid was transformed into active *E. coli* BL21 cells. After antibiotic selection and initial growth, expression was induced by IPTG. After growth, the cells were collected and lysed. The Cas12a RR protein was then enriched from the total cell protein using a Ni-NTA resin, and the Cas12a RR protein was further purified by high-performance liquid chromatography (HPLC). **(B)** Progress of protein purification is shown on a Coomassie-stained SDS-PAGE gel. M, Protein Marker; 1, Cell lysate after filtration; 2, Flow-through fluid after purification by Ni-NTA resin; 3, Buffer A eluate; 4-10, Protein eluate containing 20 mM, 50 mM, 80 mM, 100 mM, 200 mM, 250 mM, or 500 mM imidazole. **(C)** FPLC chromatogram: Representative chromatogram of Cas12a RR ion exchange (IEC). The UV absorbance in milli-arbitrary units (mAU) is plotted against the elution volume in

milliliters. The red box represents the concentrated protein fraction. **(D)** Coomassie SDS-PAGE gel staining of Cas12a RR protein after FPLC purification (Kellner et al., 2019; Wang et al., 2020).

Supplementary Figure 2

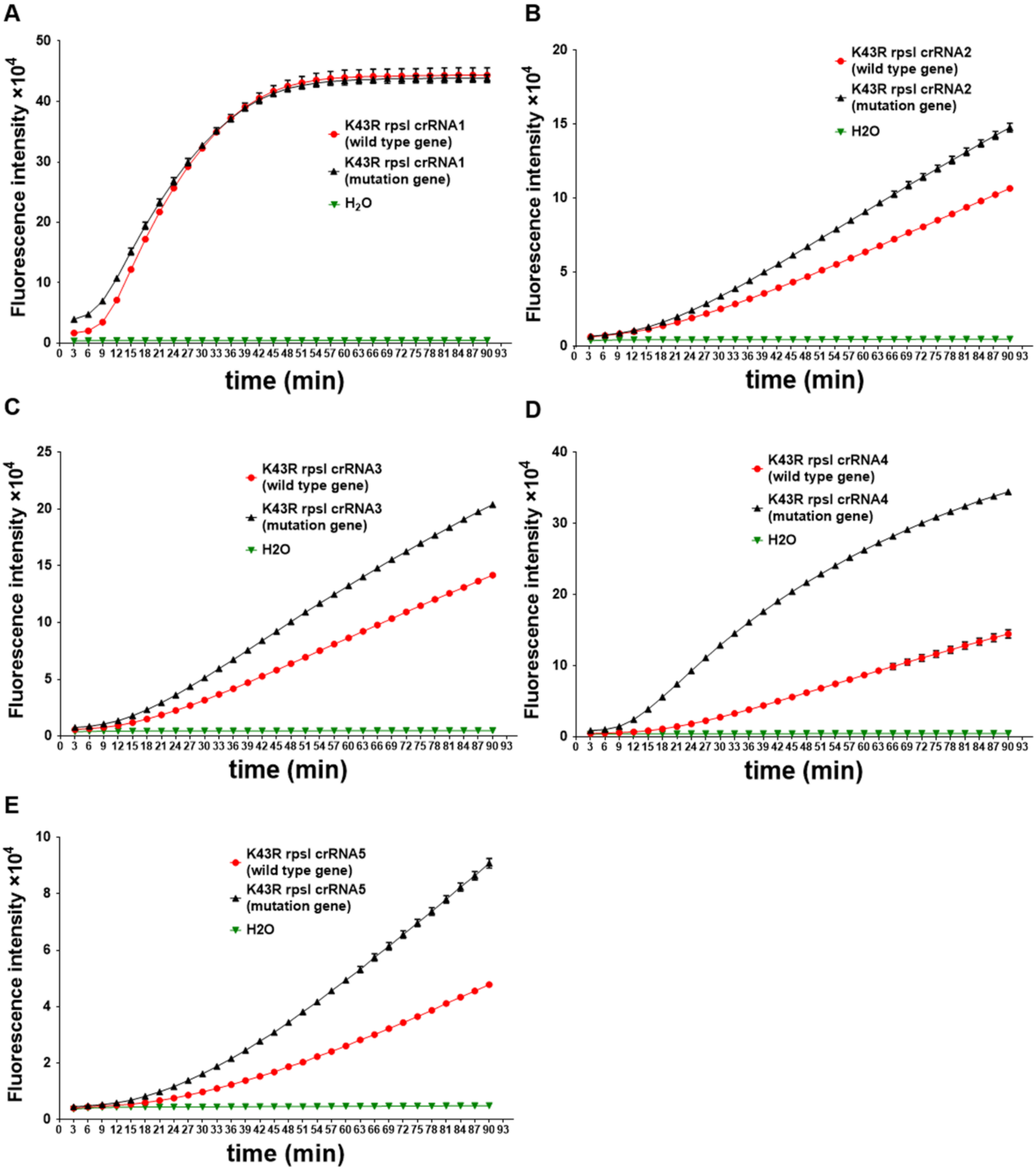

Supplementary Figure 2. Screening of the specific crRNA for the K43R mutation in the rpsl gene of STR-resistant *Mycobacterium tuberculosis*

The Cas12a RR system detects the efficiency of the K43R rpsl gene-specific crRNA1-5 of STR-resistant *M.tb*; the fluorescence intensities **(A–E)** at 90 min of the reaction are shown. The concentration of the target gene used in the above detection reaction is 1×10^{11} copies.

Supplementary Figure 3

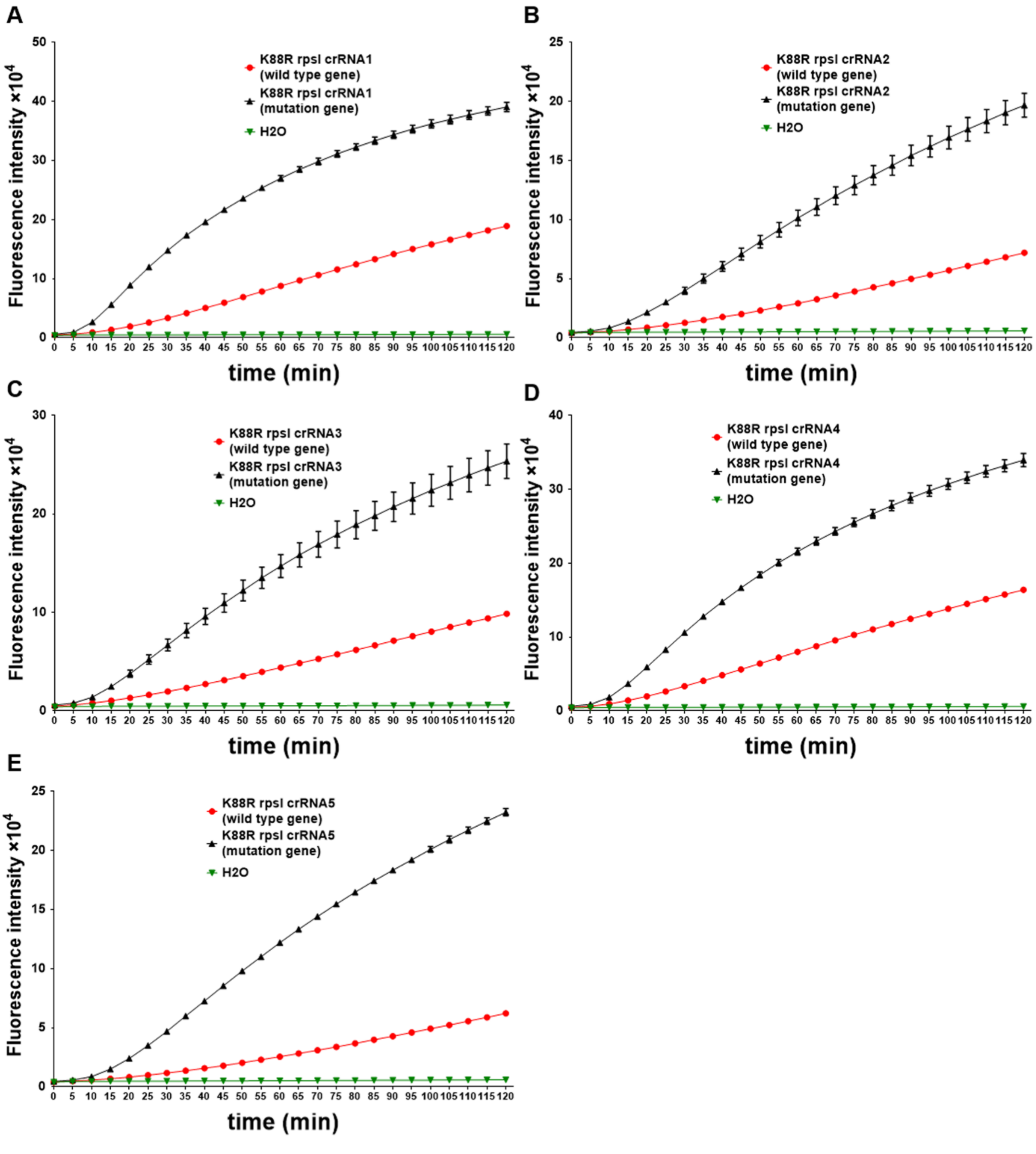

Supplementary Figure 3. Screening of the specific crRNA for the K88R mutation in the rpsl gene of STR-resistant *M. tuberculosis*

The Cas12a RR system detects the efficiency of the K88R rpsl gene-specific crRNA1-5 of STR-resistant *M.tb*; the fluorescence intensities **(A–E)** at 120 min of the reaction are shown. The concentration of the target gene used in the above detection reaction is 1×10^{11} copies.

REFERENCES

Kellner, M. J., Koob, J. G., Gootenberg, J. S., Abudayyeh, O. O., and Zhang, F. (2019). SHERLOCK: nucleic acid detection with CRISPR nucleases. Nat Protoc. 14, 2986-3012.

Wang, X., Ji, P., Fan, H., Dang, L., Wan, W., Liu, S., et al. (2020). CRISPR/Cas12a technology combined with immunochromatographic strips for portable detection of African swine fever virus. Commun Biol. 3, 62.
